# CollapsABEL: an R library for detecting compound heterozygote alleles in genome-wide association studies

**DOI:** 10.1186/s12859-016-1006-9

**Published:** 2016-04-08

**Authors:** Kaiyin Zhong, Lennart C. Karssen, Manfred Kayser, Fan Liu

**Affiliations:** Department of Genetic Identification, Erasmus University Medical Center Rotterdam, Rotterdam, The Netherlands; PolyOmica, Groningen, The Netherlands; Key Laboratory of Genomic and Precision Medicine, Beijing Institute of Genomics, Chinese Academy of Sciences, Beijing, China

**Keywords:** Genome wide association study, Next generation sequencing, Compound heterozygosity, Missing heritability

## Abstract

**Background:**

Compound Heterozygosity (CH) in classical genetics is the presence of two different recessive mutations at a particular gene locus. A relaxed form of CH alleles may account for an essential proportion of the missing heritability, i.e. heritability of phenotypes so far not accounted for by single genetic variants. Methods to detect CH-like effects in genome-wide association studies (GWAS) may facilitate explaining the missing heritability, but to our knowledge no viable software tools for this purpose are currently available.

**Results:**

In this work we present the Generalized Compound Double Heterozygosity (GCDH) test and its implementation in the R package CollapsABEL. Time-consuming procedures are optimized for computational efficiency using Java or C++. Intermediate results are stored either in an SQL database or in a so-called big.matrix file to achieve reasonable memory footprint. Our large scale simulation studies show that GCDH is capable of discovering genetic associations due to CH-like interactions with much higher power than a conventional single-SNP approach under various settings, whether the causal genetic variations are available or not. CollapsABEL provides a user-friendly pipeline for genotype collapsing, statistical testing, power estimation, type I error control and graphics generation in the R language.

**Conclusions:**

CollapsABEL provides a computationally efficient solution for screening general forms of CH alleles in densely imputed microarray or whole genome sequencing datasets. The GCDH test provides an improved power over single-SNP based methods in detecting the prevalence of CH in human complex phenotypes, offering an opportunity for tackling the missing heritability problem.

Binary and source packages of CollapsABEL are available on CRAN (https://cran.r-project.org/web/packages/CollapsABEL) and the website of the GenABEL project (http://www.genabel.org/packages).

**Electronic supplementary material:**

The online version of this article (doi:10.1186/s12859-016-1006-9) contains supplementary material, which is available to authorized users.

## Background

Compound Heterozygosity (CH) in classical genetics is the presence of two different recessive mutations at a particular gene locus, one on each chromosome [1] (Additional file 1: Figure S1). The presence of CH has been found for nearly all autosomal recessive disorders as well as other phenotypes such as red hair color [2, 3]. A relaxed form of CH, i.e., in which the genetic variants are not necessarily coding, rare, and deleterious, is likely involved in a wide range of human polygenic traits and is here referred to as generalized CH (GCH). However, individually analyzing a large number of DNA sequence variants such as single nucleotide polymorphisms (SNPs), as is the routine in genome-wide association studies (GWAS), has limited power to detect genetic associations caused by GCH. Because gene variants detected from GWAS together typically explain only a small proportion of the phenotypic variance (referred to as the “missing heritability” [4, 5]), we expect that GCH is an important source of the missing heritability.

Existing methods designed for detecting CH alleles suffer from the lack of usable implementations [6, 7] and are not suitable for the analysis of densely imputed SNP microarray data or whole genome/exome sequencing data. Previously, we have developed a collapsed double heterozygosity (CDH) test for detecting the association between CH genotypes and binary traits by applying a chi-squared statistic to pseudo-genotypes collapsed from a pair of SNPs [3], which has a sliding-window based implementation (the *cocohet* function) in the GenABEL R package [8]. CDH has been shown to have an improved power in detecting genetic association due to CH compared to the conventional single-SNP approach [3], but the previous implementation has certain limitations, including: (1) it cannot analyze quantitative traits with covariates, (2) it cannot deal with densely imputed genome data due to memory limitations, (3) computational efficiency was not optimized for large datasets, (4) lack of user-friendly interface and facilitating functions for power and type-I error estimation. These issues are solved in the current extension. Here we implement a generalized CDH (GCDH) method to overcome previous limitations and allow (1) fast analysis of densely imputed SNP data or whole genome sequencing data; (2) flexible analysis of binary and quantitative traits with covariates; (3) empirical power estimation and type-I error control; and (4) easy interface with plotting utilities. The complete analytical pipeline is implemented as an R package, called CollapsABEL, and publically available as part of the open-source collaborative GenABEL project for statistical genomics (http://www.genabel.org).

## Implementation

**Fig. 1:**
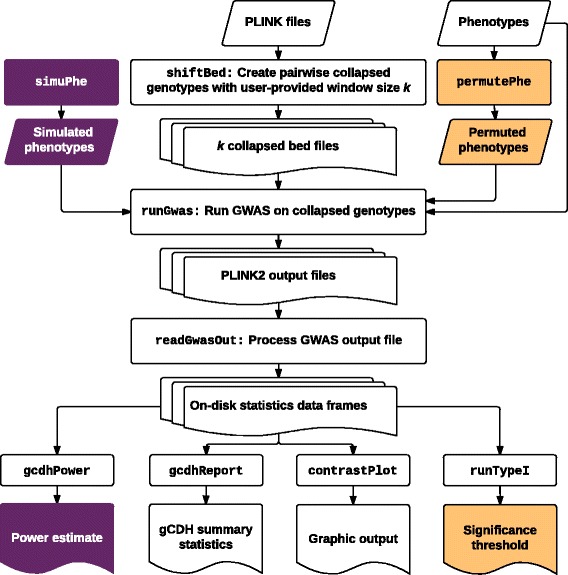
CollapsABEL flowchart

**Fig. 2:**
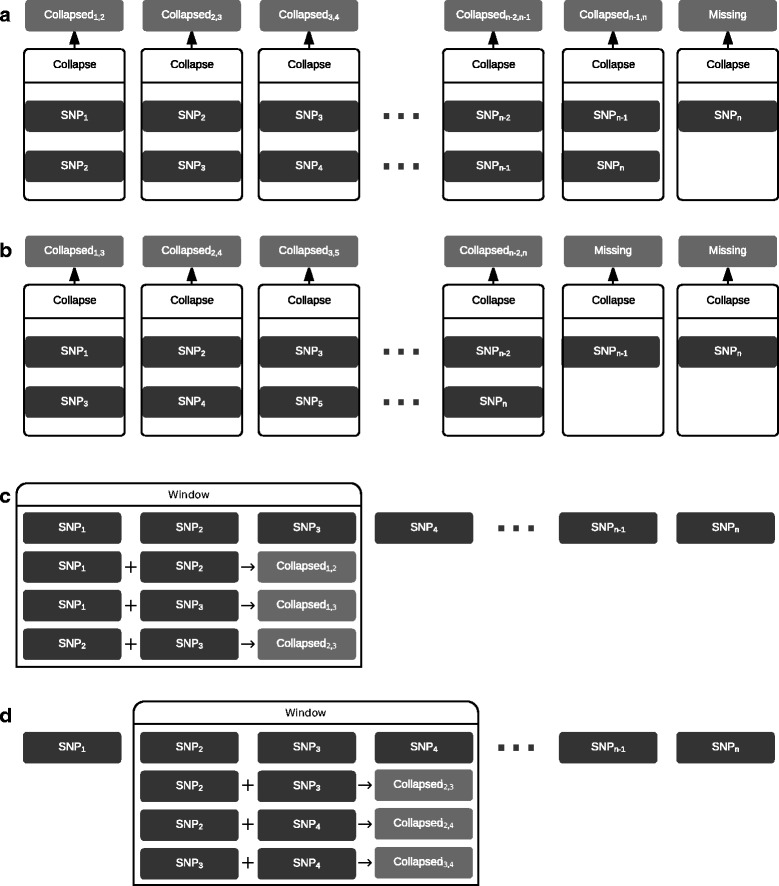
Genome-shifting algorithm compared with sliding-window algorithm. The genome-shifting algorithm starts with a PLINK binary genotype file (the bed file), and shift the whole genome one SNP at a time, each time generating a new bed file containing collapsed genotypes. The total number of new bed files is equal to the user-specified window size *k*. **a** Shift by 1 SNP. **b** Shift by 2 SNPs. The sliding-window algorithm generates collapsed genotypes for all possible combinations of SNP pairs within a window, and at each iteration slides the window forward by one SNP. **c** 1st sliding window. **d** 2nd sliding window

The analytical pipeline of CollapsABEL (with the *runGcdh* function as the main entry point), as outlined in Fig. 1, starts with the *shiftBed* function for collapsing the genotypes of a pair of SNPs according to a user provided CH model, which results in a binary coded pseudo-genotype. Considering an arbitrary pair of bi-allelic SNPs, there are 16 possible combinations, which can be organized into a 4 by 4 matrix, called the collapsing matrix. Thus we implement the genotype collapsing function *C* as a 2D array lookup function: $C\left({g}_1,{g}_2\right)={M}_{g_1,{g}_2},$ where *g*_1_, *g*_2_ are the genotype codes of the SNP pair. The default collapsing matrix (Table 1) models the scenario where the allelic effect is caused by the homozygote form of either SNP of a pair or the compound heterozygote form of two SNPs [3]. Users can also supply alternative collapsing matrices. For efficient storage of genotype data we adopt the PLINK [9] bed format, which stores each genotype into 2 bits, i.e. 4 genotypes in each byte. To speed up processing, we construct a 2D collapsing byte array from the given collapsing model and carry out the collapsing directly on pairs of bytes instead of extracting genotypes from each byte. Genotype collapsing is conducted on whole genome data using a genome-shifting algorithm (Additional file 1: Algorithm S1, an illustrative diagram is given in Fig. 2) with the function *shiftBed*. This function collapses each SNP with the *i-*th SNP downstream (*i* initialized to 1). Each window represents the scope of pairwise collapsing in one iteration, i.e. the initial SNP with *k* SNPs downstream. Therefore, for window size *k*, *shiftBed* is called *k* times to produce *k* new shifted bed files consisting of collapsed genotypes, incrementing *i* by 1 at each iteration. All functions for reading, manipulating and writing bed files call Java methods under the hood (without data copying between Java and R since the whole genome-shifting job is done in the Java Virtual Machine). Genome-shifting produces the same results as the sliding-window approach (i.e., collapsing genotypes for all pairs of SNPs within a window and then sliding over the whole genome), but is much faster for the following reasons: (1) avoidance of combinatorial calculations, (2) no duplicated computation, (3) higher throughput and fewer loops, and (4) once the collapsing matrix is given, the collapsing byte array can be generated only once, where all possible collapsing scenarios are pre-calculated according to the user-specified collapsing model and stored in a 2D array, making genotype collapsing practically as fast as array indexing, which is an *O*(1) operation.

**Table 1:** Collapsing matrices

A					
		SNP 2			
		0	1	2	3
SNP 1	0	0	0	0	0
	1	0	1	1	1
	2	0	1	0	3
	3	0	1	3	3
B					
		SNP 2			
		AA	Missing	Aa	aa
SNP 1	AA	2	2	2	2
	Missing	2	Missing	Missing	Missing
	Aa	2	Missing	2	0
	aa	2	Missing	0	0
C					
		SNP 2			
		0	1	2	3
SNP 1	0	0	0	0	0
	1	0	1	1	1
	2	0	1	0	2
	3	0	1	2	3
D					
		SNP 2			
		AA	Missing	Aa	aa
SNP 1	AA	2	2	2	2
	Missing	2	Missing	Missing	Missing
	Aa	2	Missing	2	1
	aa	2	Missing	1	0

(A) Machine representation of the default collapsing matrix. (B) Interpretation of the default collapsing matrix. Coding of input genotype follows PLINK convention, 0 (binary 00) for homozygote of minor allele, 1 (binary 01) for missing, 2 (binary 10) for heterozygote, and 3 (binary 11) for homozygote of major allele. After collapsing, the output pseudo-genotype is either 0, 2 or missing. The collapsing matrix is customizable by users, for example , an alternative collapsing matrix (C and D) will produce different pseudo-genotypes with allele coding 0, 1, 2 or missing

Once the collapsed genotypes are generated, the *runGwas* function conducts GWA scans over them by calling PLINK2 [10]. *runGwas* internally calls PLINK2 *k* times and uses linear or logistic regression models for the analysis of quantitative or binary traits, respectively, possibly also with covariates, generating *k* PLINK output files. The *readGwasOut* function then calls C++ routines for fast text processing, which loads the summary statistics from each PLINK output file, and saves these intermediate results on the hard drive in big.matrix format [11], allowing minimal RAM consumption and processing of massive datasets that would not fit in memory. Summary statistics are then extracted from these big.matrix files for both individual SNPs and collapsed genotypes of SNP pairs, which are then merged with SNP annotations and stored on the hard drive as an SQLite database (using the *gcdhReport* function), from which regions of interest can be queried without loading the whole file into memory. The *gcdhRegion* function can be used to extract regions of interest from the bed file and conduct regional GCDH analysis over it.

CollapsABEL features built-in capabilities for type-I error control and power estimation. The *runTypeI* function empirically derives the genome-wide significance threshold for GCDH by permutation analysis, i.e. the phenotype file is permuted *N* times and *N* GCDH analyses are done using these *N* permuted phenotype files, each GCDH analysis produces one global minimal *p-*value (or maximal *t* statistic), then the α quantile (or 1- α quantile of the *t* statistic) is used as the genome-wide significance threshold (which controls type-I error rate at α). The *gcdhPower* function simulates phenotypes according to user-specified allele effect sizes, range of allele frequencies and α-level, and conducts GCDH analysis on genotype data to empirically estimate the statistical power under these settings.

Statistical results can be graphically summarized by the *contrastPlot* function in the form of a contrast Manhattan plot, where *p*-values from GCDH analyses are overlaid on those from the single-SNP analysis. All plots are produced as ggplot objects [12], which can be easily customized, annotated, and exported in various image file formats.

## Results

### The Rotterdam Study

The Rotterdam Study (RS) is a prospective population-based cohort study of 14,926 participants aged 45 years and older, living in a suburb of Rotterdam, the Netherlands. Details of the study design and objectives have been described elsewhere [13]. Whole blood DNA extraction, Illumina 550–610 K genotyping, quality controls, and 1000-genomes based [14] genotype imputation have been described in detail previously [3]. After all quality controls, the current study included a total of 11,496 individuals and 15,880,747 autosomal SNPs. The Rotterdam Study has been approved by the medical ethics committee according to the Wet Bevolkingsonderzoek ERGO, executed by the Ministry of Health, Welfare and Sports of the Netherlands and all participants provided written informed consent.

### Power analysis using imputed microarray data

We conducted extensive power analyses based on SNP pairs under 50 combinations of allele effect sizes (β, varying within the range [0.5, 1.5]), minor allele frequencies (*MAF*s, varying within the range [0.01, 0.05]) and sample sizes (*N*, fixed at either 8000 or 11,000) using the Rotterdam Study imputed genetic data as the genotype pool. Under each combination of β, *MAF* and *N*, genotypes of 55 pairs of SNPs are drawn from the pool conditioned on the physical distance between each SNP of a pair (<400 kb). For each pair of SNPs (SNP a and SNP b, coded as 0, 1, or 2 minor alleles), a quantitative phenotype vector (y) is simulated according to their collapsed genotypes (x), *y* = *βx* + *ε*, *ε* ~ *N*(0, 1), where x is the collapsed genotype. Three generalized linear models are fitted using the simulated phenotype vector as dependent variable and genotypes of SNP a, genotypes of SNP b, or the collapsed genotypes as the explanatory variable. Power is calculated using *p*-value vectors from these three models under different significance thresholds (0.05, 5 × 10^-8^ or 5 × 10^-11^).

**Fig. 3:**
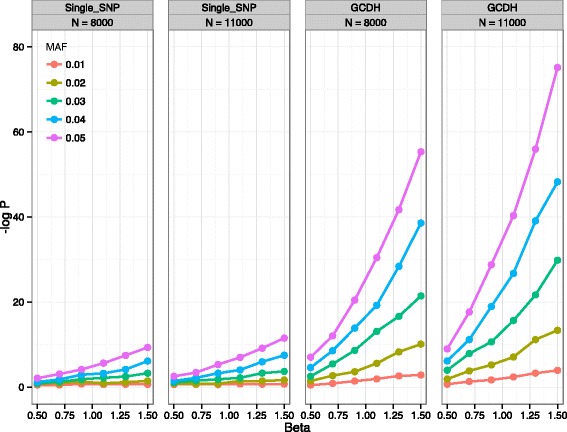
Relationship between *N, MAF, β* and median *p*-value from the GCDH analysis and single SNP association analysis. SNP pairs with different *MAFs* are drawn from 1000-Genomes imputed Rotterdam Study microarray data. Sample sizes are fixed at 8000 or 11,000. Allele effect sizes β ranges from 0.5 to 1.5. Median *p*-values for SNPs from different *MAF* groups are distinguished using different colors. In total 2750 simulations are conducted

In every category of *N*, *MAF*, and *β*, *p*-values from GCDH are consistently more significant than those from single-SNP approach (Fig. 3); GCDH also has higher power than the single-SNP approach (e.g. when *N* = 8000, *β* = 1.1, power of single-SNP analysis is 0 % while that of GCDH is 98 %). Even when we use a much more stringent threshold of 5 × 10^-11^, GCDH is still more powerful compared with using 5 × 10^-8^ as the threshold for the single SNP approach (e.g. when *N* = 8000, *β* = 1.3, power of single-SNP analysis is 0 % while that of GCDH is 96 %). Power of GCDH increases with *MAF*, *N* and *β*; for example, if we fix *N* = 8000, *β* = 0.9, then as *MAF* changes from 0.01 to 0.05, the power of GCDH increases from 0 to 100 %. When *β* = 0.09, *MAF* = 0.03 and *N* increases from 8000 to 11,000, power of GCDH increases from 53 to 85 %, and when *N* = 11,000, *MAF* = 0.04, and *β* changes from 0.5 to 1.5, power of GCDH increases from 27 to 100 % (Table 2). Similar observations can be made when using the more stringent 5 × 10^-11^ threshold.

**Table 2:** Power analysis using simulated phenotypes and SNP pairs randomly selected from the Rotterdam Study

			Threshold 5 × 10^-2^			Threshold 5 × 10^-8^			Threshold 5 × 10^-11^
*N*	*β*	*MAF*	a	b	GCDH	a	b	GCDH	GCDH
8000	0.50	0.01	0.05	0.05	0.24	0.00	0.00	0.00	0.00
8000	0.50	0.02	0.09	0.09	0.53	0.00	0.00	0.00	0.00
8000	0.50	0.03	0.20	0.13	0.84	0.00	0.00	0.04	0.00
8000	0.50	0.04	0.31	0.40	1.00	0.00	0.00	0.22	0.04
8000	0.50	0.05	0.42	0.45	1.00	0.00	0.00	0.40	0.15
8000	0.70	0.01	0.04	0.09	0.45	0.00	0.00	0.00	0.00
8000	0.70	0.02	0.11	0.09	0.84	0.00	0.00	0.02	0.00
8000	0.70	0.03	0.18	0.29	1.00	0.00	0.00	0.25	0.04
8000	0.70	0.04	0.47	0.45	1.00	0.00	0.00	0.65	0.33
8000	0.70	0.05	0.67	0.75	1.00	0.00	0.00	0.95	0.76
8000	0.90	0.01	0.05	0.09	0.31	0.00	0.00	0.00	0.00
8000	0.90	0.02	0.20	0.15	0.96	0.00	0.00	0.09	0.00
8000	0.90	0.03	0.49	0.36	1.00	0.00	0.00	0.53	0.20
8000	0.90	0.04	0.56	0.82	1.00	0.02	0.02	0.98	0.84
8000	0.90	0.05	0.87	0.93	1.00	0.06	0.02	1.00	0.98
8000	1.10	0.01	0.02	0.09	0.67	0.00	0.00	0.00	0.00
8000	1.10	0.02	0.27	0.18	1.00	0.00	0.00	0.27	0.07
8000	1.10	0.03	0.57	0.61	1.00	0.00	0.00	0.98	0.78
8000	1.10	0.04	0.80	0.84	1.00	0.02	0.00	1.00	1.00
8000	1.10	0.05	1.00	0.98	1.00	0.06	0.15	1.00	1.00
8000	1.30	0.01	0.07	0.04	0.85	0.00	0.00	0.00	0.00
8000	1.30	0.02	0.25	0.31	1.00	0.00	0.00	0.44	0.20
8000	1.30	0.03	0.62	0.67	1.00	0.00	0.00	1.00	0.96
8000	1.30	0.04	0.89	0.93	1.00	0.02	0.00	1.00	1.00
8000	1.30	0.05	1.00	0.98	1.00	0.33	0.36	1.00	1.00
8000	1.50	0.01	0.13	0.09	0.85	0.00	0.00	0.07	0.00
8000	1.50	0.02	0.25	0.40	1.00	0.00	0.00	0.76	0.35
8000	1.50	0.03	0.78	0.78	1.00	0.00	0.00	1.00	1.00
8000	1.50	0.04	0.96	1.00	1.00	0.13	0.13	1.00	1.00
8000	1.50	0.05	1.00	1.00	1.00	0.39	0.59	1.00	1.00
11000	0.50	0.01	0.00	0.05	0.27	0.00	0.00	0.00	0.00
11000	0.50	0.02	0.04	0.15	0.65	0.00	0.00	0.00	0.00
11000	0.50	0.03	0.18	0.20	0.96	0.00	0.00	0.00	0.00
11000	0.50	0.04	0.49	0.35	1.00	0.00	0.00	0.27	0.05
11000	0.50	0.05	0.56	0.60	1.00	0.00	0.00	0.78	0.44
11000	0.70	0.01	0.04	0.04	0.53	0.00	0.00	0.02	0.00
11000	0.70	0.02	0.11	0.20	0.89	0.00	0.00	0.07	0.00
11000	0.70	0.03	0.35	0.29	1.00	0.00	0.00	0.42	0.07
11000	0.70	0.04	0.51	0.62	1.00	0.00	0.00	0.95	0.76
11000	0.70	0.05	0.87	0.87	1.00	0.02	0.02	1.00	0.98
11000	0.90	0.01	0.02	0.04	0.55	0.00	0.00	0.00	0.00
11000	0.90	0.02	0.20	0.35	1.00	0.00	0.00	0.33	0.09
11000	0.90	0.03	0.49	0.40	1.00	0.00	0.00	0.85	0.56
11000	0.90	0.04	0.87	0.75	1.00	0.00	0.00	1.00	0.96
11000	0.90	0.05	0.96	0.98	1.00	0.02	0.05	1.00	1.00
11000	1.10	0.01	0.13	0.05	0.76	0.00	0.00	0.00	0.00
11000	1.10	0.02	0.35	0.27	1.00	0.00	0.00	0.58	0.20
11000	1.10	0.03	0.53	0.56	1.00	0.00	0.00	1.00	0.91
11000	1.10	0.04	0.93	0.91	1.00	0.02	0.02	1.00	1.00
11000	1.10	0.05	1.00	1.00	1.00	0.27	0.24	1.00	1.00
11000	1.30	0.01	0.13	0.07	0.91	0.00	0.00	0.02	0.00
11000	1.30	0.02	0.41	0.46	1.00	0.00	0.00	0.91	0.61
11000	1.30	0.03	0.76	0.78	1.00	0.00	0.00	1.00	1.00
11000	1.30	0.04	0.98	1.00	1.00	0.11	0.16	1.00	1.00
11000	1.30	0.05	1.00	1.00	1.00	0.55	0.62	1.00	1.00
11000	1.50	0.01	0.04	0.11	0.87	0.00	0.00	0.04	0.00
11000	1.50	0.02	0.47	0.44	1.00	0.00	0.00	0.96	0.76
11000	1.50	0.03	0.89	0.91	1.00	0.02	0.05	1.00	1.00
11000	1.50	0.04	0.98	0.96	1.00	0.18	0.24	1.00	1.00
11000	1.50	0.05	1.00	1.00	1.00	0.85	0.87	1.00	1.00

a: Power estimates for causal SNP a

b: Power estimates for causal SNP b

*N*: Sample size

GCDH: Power estimates for the collapsed genotypes of a and b

*β*: Coefficient used for simulation of phenotypes

### Power analysis using whole exome-sequencing data

In the Rotterdam Study whole exome-sequencing data were available for 1037 individuals and 167,209 coding variants. Regions of width 10 to 50 kb are drawn randomly, and in each region two SNPs satisfying certain *MAF* criteria (5 strata ranging from 0.0015 to 0.1) are randomly set as causal, a phenotype is simulated according to the collapsed genotype model as described above. Due to the small sample size in the exome-sequencing data, we take larger effect sizes (β ranging from 1 to 4) and *MAF*s (ranging from 0.0015 to 0.1) to demonstrate the differences between GCDH and single-SNP approach. Additionally, the two causal SNPs are selected to be in low LD (*r*^2^ < 0.01) assuming that they are on different haplotypes, otherwise the region is discarded a new one is drawn. These two causal SNPs are included or excluded depending on the purpose of the analysis (see below). A null-phenotype consisting of only a standard normal noise term is also simulated for the purpose of monitoring the null distribution of the test statistics and controlling for Type-I error.

For each region, two single-SNP GWA and two GCDH scans are done, one using the associated phenotype and the other using the null-phenotype, both with window size set at 50 SNPs. In each scan using the associated phenotype, we obtain two regional minimal *p* values, one with the results from causal SNPs included, one with them excluded. Thus each loop generates six *p* values: *P*_*s*,*d*_ (single-SNP approach with dummy phenotype), *P*_*g*,*d*_ (GCDH with dummy phenotype), *P*_*s*_ (single-SNP approach, causal SNPs genotyped), *P*_*g*_ (GCDH, causal SNPs genotyped), *P*_*s*,*n*_ (single-SNP approach, causal SNPs not genotyped), *P*_*g*,*n*_ (GCDH, causal SNPs not genotyped). In total 10,000 such scans are conducted according to different combinations of effect size and *MAF* interval, around 500 loops for each combination. At the end of the simulation, we obtained six vectors of *p*-values, *P*_*s*,*d*_, *P*_*g*,*d*_, *P*_*s*_, *P*_*g*_, *P*_*s*,*n*_, *P*_*g*,*n*_, each of length 10,000. For each of *P*_*s*,*d*_ and *P*_*g*,*d*_ we derived the 5 % quantile, which represents two thresholds *T*_*s*_ and *T*_*g*_ under the null (and thus controls type-I error rate at 5 %). The power of single-SNP approach is the proportion of *p*-values in *P*_*s*_ that are smaller than *T*_*s*_ and the power of GCDH is the proportion of *p*-values in *P*_*g*_ which are smaller than *T*_*s*_. Similarly, power estimations of single-SNP approach and GCDH when causal SNPs are excluded from the region, i.e. assuming causal SNPs untyped, are derived from *P*_*s*,*n*_ and *P*_*g*,*n*_.

The results clearly demonstrated that when *MAF* is above 0.02, GCDH has consistently higher power than the single-SNP approach, whether the causal SNPs are present or not. For example, when *MAF* is between 0.04 and 0.06, *β* = 2, power of single-SNP method is 0.28 while that of GCDH is 0.69; when the causal SNPs are untyped, these numbers drop to 0.18 and 0.32 respectively, still giving a nearly two-fold increase in GCDH. Similar to what we observed in the simulation done with imputed microarray data, power of GCDH increases with *β* and *MAF*, for instance, when *β* = 2 and *MAF* interval changes from (0, 0.02) to (0.08, 0.1), power of GCDH changes from 16 to 83 % when causal SNPs are typed and from 11 % to 46 % when they are not (Table 3).

**Table 3:** Comparison of power between GCDH and single-SNP approaches in analysis of exome-sequencing data from the Rotterdam Study

		Causal SNPs available		Causal SNPs excluded	
*β*	*MAF*	Single-SNP	GCDH	Single-SNP	GCDH
1	(0.00, 0.02)	0.09	0.09	0.08	0.08
1	(0.02, 0.04)	0.17	0.23	0.13	0.14
1	(0.04, 0.06)	0.18	0.28	0.11	0.15
1	(0.06, 0.08)	0.29	0.43	0.19	0.22
1	(0.08, 0.10)	0.20	0.44	0.14	0.18
2	(0.00, 0.02)	0.13	0.16	0.10	0.11
2	(0.02, 0.04)	0.29	0.56	0.19	0.29
2	(0.04, 0.06)	0.28	0.69	0.18	0.32
2	(0.06, 0.08)	0.41	0.75	0.31	0.41
2	(0.08, 0.10)	0.41	0.83	0.28	0.46
3	(0.00, 0.02)	0.18	0.19	0.12	0.12
3	(0.02, 0.04)	0.47	0.72	0.32	0.41
3	(0.04, 0.06)	0.45	0.86	0.32	0.49
3	(0.06, 0.08)	0.55	0.89	0.41	0.58
3	(0.08, 0.10)	0.65	0.94	0.46	0.63
4	(0.00, 0.02)	0.23	0.26	0.13	0.15
4	(0.02, 0.04)	0.55	0.83	0.41	0.53
4	(0.04, 0.06)	0.56	0.94	0.43	0.62
4	(0.06, 0.08)	0.70	0.97	0.54	0.71
4	(0.08, 0.10)	0.75	0.98	0.58	0.76

The simulation analyses are conducted based on the exom sequencing data from Rotterdam Study 1 (RS1), consisting of 1037 individuals and 167,209 SNPs. Power estimates are calculated from 10,000 simulations. Type-I error rate for single-SNP and GCDH analyses are controlled at 5 %

### Example GCDH analysis using a simulated phenotype

**Fig. 4:**
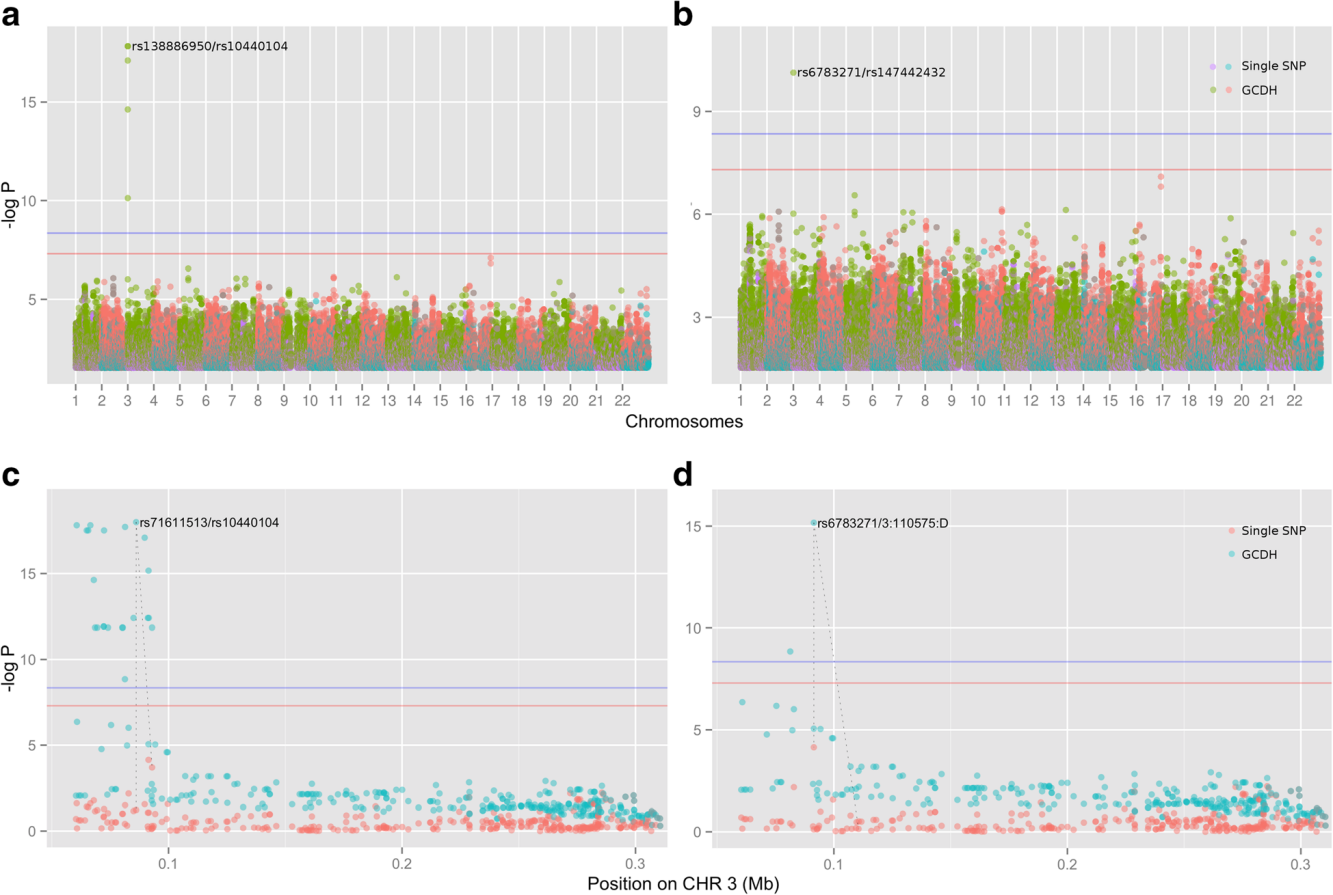
GCDH analysis using a simulated phenotype. Genotype data is from Rotterdam Study (11,496 subjects and 2,744,740 SNPs after setting *MAF* in the interval [0.01, 0.1], and only keeping SNPs that are genotyped in every subject). Phenotype is simulated with effect size 0.7 plus a random error term from the standard normal distribution according to the collapsed genotype of two randomly selected SNPs (rs138886950 and rs10440104 in this case), and run GCDH using this as the phenotype. Genome-wide significance threshold (the red horizontal line in the figure) is set at 5.0 × 10^-8^ for the single-SNP approach, for GCDH (the blue horizontal line) it is set empirically at 4.5 × 10^-9^ by permutation analysis (see the runTypeI function in CollapsABEL). Window size is set 55. **a** Genome-wide scan with causal SNPs available. **b** Genome-wide scan without genotypes of causal SNPs. **c** Regional GCDH with causal SNPs available. **d** Regional GCDH without genotypes of causal SNPs

As a demonstration, we simulate a phenotype (effect size 0.7 plus a random error term from the standard normal distribution) according to the collapsed genotype of two randomly selected two causal SNPs (rs138886950 and rs10440104 on 3p25.3). We conducted a GCDH analysis with window size set at 55 and *p*-value filter set at 0.03, so that a total of 80,172 SNPs were included. When the causal SNPs are available, GCDH detects strong signals in and only in the corresponding region (around 0.1 Mb on chromosome 3, best *p*-value 1.52 × 10^-18^ from rs138886950/rs10440104 Fig. 4a), while the single-SNP approach fails to do so (best *p*-value 8.44 × 10^-7^ from rs116605385, Fig. 4a). When the two causal SNPs are removed from the analysis, the genome-wide GCDH scan still picks-up the correct locus, although with less significant *p-*values (best *p*-value 7.52 × 10^-11^ from rs6783271/rs147442432, Fig. 4b), and single SNP analysis gives the same result as before.

We further conducted a regional GCDH analysis using all imputed SNPs within 250 kb flanking rs138886950, including 324 SNPs in total. The regional scan’s signal still weakens when causal SNPs are untyped, but is much more significant than what we got from the genome-wide scan (best *p*-value: 9.96 × 10^-19^ from rs71611513/rs10440104 when the causal SNPs are typed, and 6.83 × 10^-16^ from rs6783271/3:110575:D when they are not typed, Fig. 4c and d). This is because in the genome-wide scan we applied a fairly aggressive *p*-value filter here (0.03), i.e. all SNPs that do not pass the association test with a *p*-value more significant than 0.03 are filtered out. This example clearly illustrates that applying a *p*-value filter could remove a considerable number of SNPs that individually are not significantly associated with the phenotype but may be so when collapsed with other SNPs.

Using the top 6 pairs of SNPs from the genome-wide scan, GCDH can explain 1.61 % of the phenotypic variation, while the single-SNP method only explains 0.39 % (Table 4).

**Table 4:** Percentage of variation explained with GCDH or single-SNP method using simulated phenotype

SNP	*R* ^2^ GCDH (%)	*R* ^2^ Single-SNP (%)
rs797501	0.67	0.01
rs10886810	0.26	0.06
rs10514590	0.24	0.07
rs111600221	0.20	0.07
rs6783271	0.20	0.04
rs138886950	0.04	0.14
Total	1.61	0.39

### Performance and memory consumption

Using a segment from the RS genotype data consisting of 13,500 SNPs and 2693 individuals, we measured the time and space performance of the *runGcdh* function on a MacBook Pro with 2.3 GHz Intel Core i7-4850HQ and 16 GB 1600 MHz DDR3 RAM (Table 5). Running time goes up linearly as window size increases, and given a dataset with 10 million SNPs and 10,000 individuals, it is estimated to take about 130 h, which agrees with our experience in practice. Memory consumption grows much slower than running time—in this benchmark, when window size was increased from 10 to 300 (by 2900 %), RAM usage grew by only 18 %.

**Table 5:** Benchmarks of the *runGcdh* function using a dataset of 13,500 SNPs and 2693 individuals and a simulated phenotype

Window size	Time (seconds)	RAM used (MB)
10	20	205
20	36	224
30	54	223
100	174	233
200	344	238
300	514	242

## Discussion

CollapsABEL offers an increased power in detecting genetic associations caused by CH-like interactions compared to traditional single SNP-based GWA approach. Computational efficiency of our method is optimized by (1) using Java and C++ for critical tasks, (2) using genome-shifting algorithm for high-throughput genotype collapsing, and (3) using the already optimized PLINK2 for statistical tests. The computational burden may be greatly reduced by applying a *p*-value filter in the initial scan to keep only the SNPs with some marginal effects. However, in our simulation example we illustrate that this filter should be used with caution, as marginally non-significant SNPs may be highly significant when interacting with other SNPs in the CH form. This also implies that CollapsABEL has a high potential in helping to solve the missing heritability problem currently faced in gene mapping of complex traits. We recommend not using *p*-value filter, but when computational time is expected to be too high, a *p*-value filter can be useful (e.g. filter by *p* < 0.1 will roughly result in a 90 % reduction in running time).

CollapsABEL operates on PLINK bed files with Java file streams and therefore can deal with large datasets that do not fit into RAM. Pairwise genotype collapsing does potentially introduce a challenging computational burden, depending on the parameters used. Window size is a major contributor to computational burden; it should be just large enough to cover the desired range in terms of base pairs, which depends on the genotype density used. *MAF* is an important factor affecting whether any positive collapsed genotype can be found at all; our simulation studies clearly demonstrated that the GCDH method has limited power when the *MAF* is less than 1 % for relative large sample sizes (*N* = 11,000, Fig. 3).

There has been some software tools developed for epistasis analysis, such as BiForce [15], iLoci [16], BOOST [17], SNPHarvester [18], and SNPRuler [19]. These tools have been highly optimized to handle substantial computational burden and often use novel screening methods to reduce the number of pairwise interactions to be tested. However, these tools are designed to detect general forms of SNP interactions at the genome-wide level in a pair-wise manner, while CollapsABEL focuses on detecting GCH in each genomic region using a sliding window approach. Therefore, these previously developed tools are not directly comparable with CollapsABEL considering that their analysis scope and targets, total numbers of tests, and levels of type-I error rate are substantially different.

Future improvements of CollapsABEL will focus on (1) Dynamic window-size determination using a user-supplied base-pair range (currently implemented as a fixed number of SNPs); (2) Deriving genome-wide type-I error thresholds analytically. Currently we use $\frac{5\times {10}^{-8}}{k}$ as threshold for genome-wide significance and use permutation analysis to empirically estimate type-I error rates, the threshold is conservative and permutation analysis is overly time-consuming; (3) Better handling missing values; (4) Customizable filter function. Currently we allow users to filter SNPs by providing a threshold for marginal *p*-values, this can be generalized to a function that returns a Boolean, so that users can choose SNPs based on any criteria; (5) Analysis of related subjects. At the moment population substructure can be adjusted by using genetic principle components as covariates, we plan to include mixed models in a future version. Some limitations will persist, though: (1) The GCDH approach identifies pairs of SNPs in the discovery cohort, the chance of finding both exact SNPs in other replication cohorts is smaller than finding only one SNP as required in conventional GWAS; this makes it more difficult for exact replication studies and meta-analysis of GWAS results; (2) The current implementation is ignorant of the scenario where CH-like association involves more than 2 causal variants because considering higher order interactions will quickly overload the capacity of existing supercomputing facilities. However, in our extensive simulations, we found that when multiple such variants exist, using an alternative collapsing matrix (additive model, Table 1C and D) instead of the default one (recessive model) has improved power in finding the CH-like association caused by multiple variants. This is likely explained by the fact that the noted pair of interacting variants is additionally in compound heterozygote with some other unknown variants. In our recent GWAS of perceived age [20], we found that testing a compound marker collapsed from four missense variants in the *MC1R* gene resulted in a drastically improved association signal (*p* = 2.7 × 10^-12^) than testing the four variants individually (min *p* = 10^-6^). Applying CollapsABEL to the perceived age dataset using the alternative collapsing matrix identifies the MC1R locus with a genome-wide significant signal as expected (data not shown), even though only pairwise collapsing was performed. Therefore, in case of the presence of multiple CH alleles we recommend to first run the initial scan using the alternative collapsing matrix and then conduct higher order interaction analysis only in the promising regions. We plan to add regional analysis functions for this in the next versions; (3) GCH still has a limited power to detect rare causal variants (MAF < 1 %) as demonstrated by our simulations which can only be overcome by extremely large sample sizes (typically > 100,000).

## Conclusions

CollapsABEL is powerful, flexible, and computationally efficient for detecting GCH in genome-wide association studies using (imputed) SNP microarray data or whole genome/whole exome sequencing studies. CollapsABEL may help finding novel gene variants that explain additional proportions of the missing heritability for a wide range of human complex traits and diseases.

## Availability and requirements

Project name: CollapsABEL

Availability: http://www.genabel.org; https://cran.r-project.org/web/packages/CollapsABEL

Operating systems: Linux/Mac OS X (Tested on Ubuntu 14.04)

Programming languages: R, Java, and C++

Other requirements: Java 1.8+, PLINK2

License: GNU GPLv3

Any restrictions to use by non-academics: GNU GPLv3

The source package CollapsABEL and its auxiliary package collUtils (compress into Additional file 2, please unzip before install) are also submitted along with this manuscript. Installation guide and sample code using a simulated dataset with 13500 SNPs and 2693 individuals are provided in Additional file 3.

## Additional files

Additional file 1: Diagram for CH and Pseudo-code for the genome-shifting algorithm. (DOCX 667 kb) Additional file 2: Source code of CollapsABEL the auxiliary package collUtils including documentation. (GZ 6805 kb) Additional file 3: Installation guide for CollapsABEL and sample code using a simulated dataset with 13500 SNPs and 2693 individuals. (PDF 900 kb)
