# Supplementary material for: CollapsABEL: an R library for detecting compound heterozygote alleles in genome-wide association studies
Source: BMC Bioinformatics. 2016 Apr 8;17:156. doi: 10.1186/s12859-016-1006-9 (PMC4826552; doi:10.1186/s12859-016-1006-9)
Supplement: Additional file 1: — Diagram for CH and Pseudo-code for the genome-shifting algorithm. (DOCX 667 kb) [file 12859_2016_1006_MOESM1_ESM.docx]

**Figure S1.** Illustration of compound heterozygosity. (A) The state of possessing two identical mutations in a particular gene (homozygosity) may lead to a recessive phenotype. (B) The presence of two different mutations at the same gene locus, both inside coding regions, one on each chromosome (compound heterozygote), may lead to the same recessive phenotype. (C) An example of generalized CH, where the second mutation occurs inside a non-coding region.

**
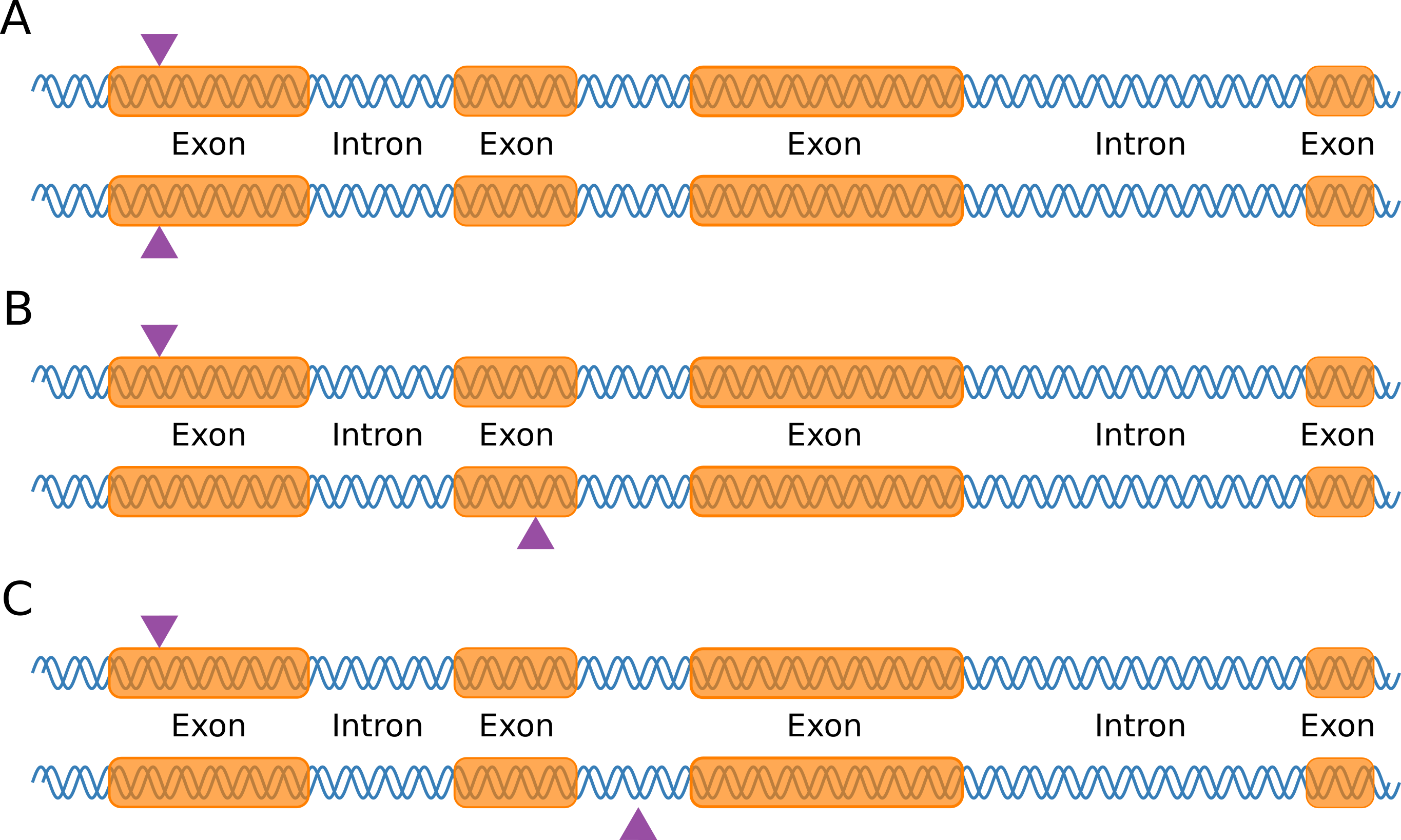
**

**Algorithm S1. G**enome shifting algorithm.

| Input: bed_file_in (input bed file), k (window size)  Output: bed_file_out_1, bed_file_out_2, ..., bed_file_out_k |
| --- |
| 01: for each i in {sequence from 1 to k}:  02: stream1 <- open a reading stream on bed_file_in  03: stream2 <- open a reading stream on bed_file_in  04: stream1 skip the first 3 bytes  05: stream2 skip the first (3 + i * (number of bytes per SNP)) bytes  06: stream3 <- open a writing stream for bed_file_out_i  07: for each j in {sequence from 0 to (total number of SNPs - 1)}:  08: buffer_collapsed <- initialize a new byte array  09: buffer1 <- read genotypes of one SNP from stream1  10: if j >= (number_of_SNPs - i):  11: fill buffer_collapsed with NA  12: else:  13: buffer2 <- read genotype of one SNP from stream2  14: for each genotypes_byte1 in buffer1, each genotypes_byte2 in buffer2:  15: coerce genotypes_byte1 and genotypes_byte2 into integers  16: collapsed_byte <- look up collapsing_byte_array at coordinates (genotypes_byte1, genotypes_byte2)  17: add collapsed_byte to buffer_collapsed  18: end for  19: end if  20: write buffer_collapsed to stream3  21: end for  22: close all streams  23: end for |
