# Supplementary material for: CollapsABEL: an R library for detecting compound heterozygote alleles in genome-wide association studies
Source: BMC Bioinformatics. 2016 Apr 8;17:156. doi: 10.1186/s12859-016-1006-9 (PMC4826552; doi:10.1186/s12859-016-1006-9)
Supplement: Additional file 3: — Installation guide for CollapsABEL and sample code using a simulated dataset with 13500 SNPs and 2693 individuals. (PDF 900 kb) [file 12859_2016_1006_MOESM3_ESM.pdf]

Getting started with CollapsABEL

This documents describes installation of the CollapsABEL package and provides a small example. The code here has been tested on a machine with Ubuntu 14.04 64 bit. Similar procedure can be adapted to Mac OS X. The currently version of CollapsABEL does not support Windows.

Installation

Install R and other dependencies

To use the latest version of R, edit /etc/apt/sources.list and add the line:

```
deb http://cran-mirror.cs.uu.nl/bin/linux/ubuntu trusty/
```

Change trusty to your Ubuntu version, of course. You can also use a different mirror site. Then you can update the list of available software. In terminal:

```
sudo apt-get update
```

If you get an error similar to the following:

```
W: GPG error: http://cran-mirror.cs.uu.nl trusty/ Release: The following signatures couldn't be verified because the
public key is not available: NO_PUBKEY 51716619E084DAB9
```

You need to import the gpg public key. In terminal:

```
sudo apt-key adv --keyserver keyserver.ubuntu.com --recv-keys 51716619E084DAB9
```

Just replace the key with what you see in your error message. Now you can install R. In terminal:

```
sudo apt-get update && sudo apt-get  install libcurl4-openssl-dev libxml2 r-base wget liblzma-dev
```

Set up Java

Install Oracle JDK 1.8:

In terminal:

```
sudo add-apt-repository ppa:webupd8team/java -y
sudo apt-get update
sudo apt-get install oracle-java8-installer oracle-java8-set-default
```

Configure Java with R:

In terminal:

```
sudo R CMD javareconf
```

The above command should be able to recognize the Java installation on your system, and should emit some message like this:

```
Java interpreter : /usr/bin/java
Java version     : 1.8.0_60
Java home path   : /usr/lib/jvm/java-8-oracle/jre
Java compiler    : /usr/bin/javac
Java headers gen.: /usr/bin/javah
Java archive tool: /usr/bin/jar
trying to compile and link a JNI program
detected JNI cpp flags    : -I$(JAVA_HOME)/../include -I$(JAVA_HOME)/../include/linux
detected JNI linker flags : -L$(JAVA_HOME)/lib/amd64/server -ljvm
gcc -std=gnu99 -I/usr/share/R/include -DNDEBUG -I/usr/lib/jvm/java-8-oracle/jre/../include -I/usr/lib/jvm/java-8-
oracle/jre/../include/linux     -fpic  -g -O2 -fstack-protector-strong -Wformat -Werror=format-security -
D_FORTIFY_SOURCE=2 -g  -c conftest.c -o conftest.o
gcc -std=gnu99 -shared -L/usr/lib/R/lib -Wl,-Bsymbolic-functions -Wl,-z,relro -o conftest.so conftest.o -
L/usr/lib/jvm/java-8-oracle/jre/lib/amd64/server -ljvm -L/usr/lib/R/lib -lR
JAVA_HOME        : /usr/lib/jvm/java-8-oracle/jre
Java library path: $(JAVA_HOME)/lib/amd64/server
...
```

Install PLINK2

Go to PLINK2 website and download the package, extract it and put the `plink` executable in your `$PATH`, `/usr/local/bin`, for example (if you are the administrator of the machine).

Install R packages

In R:

```
# dependencies
install.packages( c("rJava"), repos = "http://cran.us.r-project.org")
install.packages( c("Rcpp"), repos = "http://cran.us.r-project.org")
install.packages( c("R.utils"), repos = "http://cran.us.r-project.org")
install.packages( c("RSQLite"), repos = "http://cran.us.r-project.org")
install.packages( c("dplyr", "ggplot2", "stringr"), repos = "http://cran.us.r-project.org")
install.packages( c("biganalytics", "bigmemory"), repos = "http://cran.us.r-project.org")
# replace the paths
install.packages(c("collUtils_1.0.4.tar.gz", "CollapsABEL_0.10.6.tar.gz"), type = "source", repos = NULL)
```

Run CollapsABEL with a toy example

Download the dataset

In terminal:

```
cd /tmp
wget https://bitbucket.org/kindlychung/collapsabel/downloads/testfiles.zip
unzip -o testfiles.zip
rm testfiles.zip
cd CollapsABEL_test
```

Load package and setup the GWAS object

In R:

```
library(CollapsABEL)
rbed_info = rbedInfo(bedstem = "test", TRUE)
pl_gwas = plGwas(rbed_info,
        pheno = "test.phe",
        pheno_name = "y",
        covar_name = "sex,age",
        gwas_tag = "test_y_sex_age")
```

Run GCDH, window size set at 5

```
gcdh_res = runGcdh(pl_gwas, n_shift = 5)
```

Read results from database

```
gcdh_report = getQuery(gcdhReport(gcdh_res), "select * from gcdh_report")
head(gcdh_report)
```

Manhattan plot of single-SNP results

```
mydata = manhattanData(gcdh_report$CHR1, gcdh_report$BP1, gcdh_report$P1, gcdh_report$SNP1)
myplot = manhattanPlot(mydata)
library(ggplot2)
ggsave("/tmp/mplot.png", myplot, width = 10, height = 5)
```

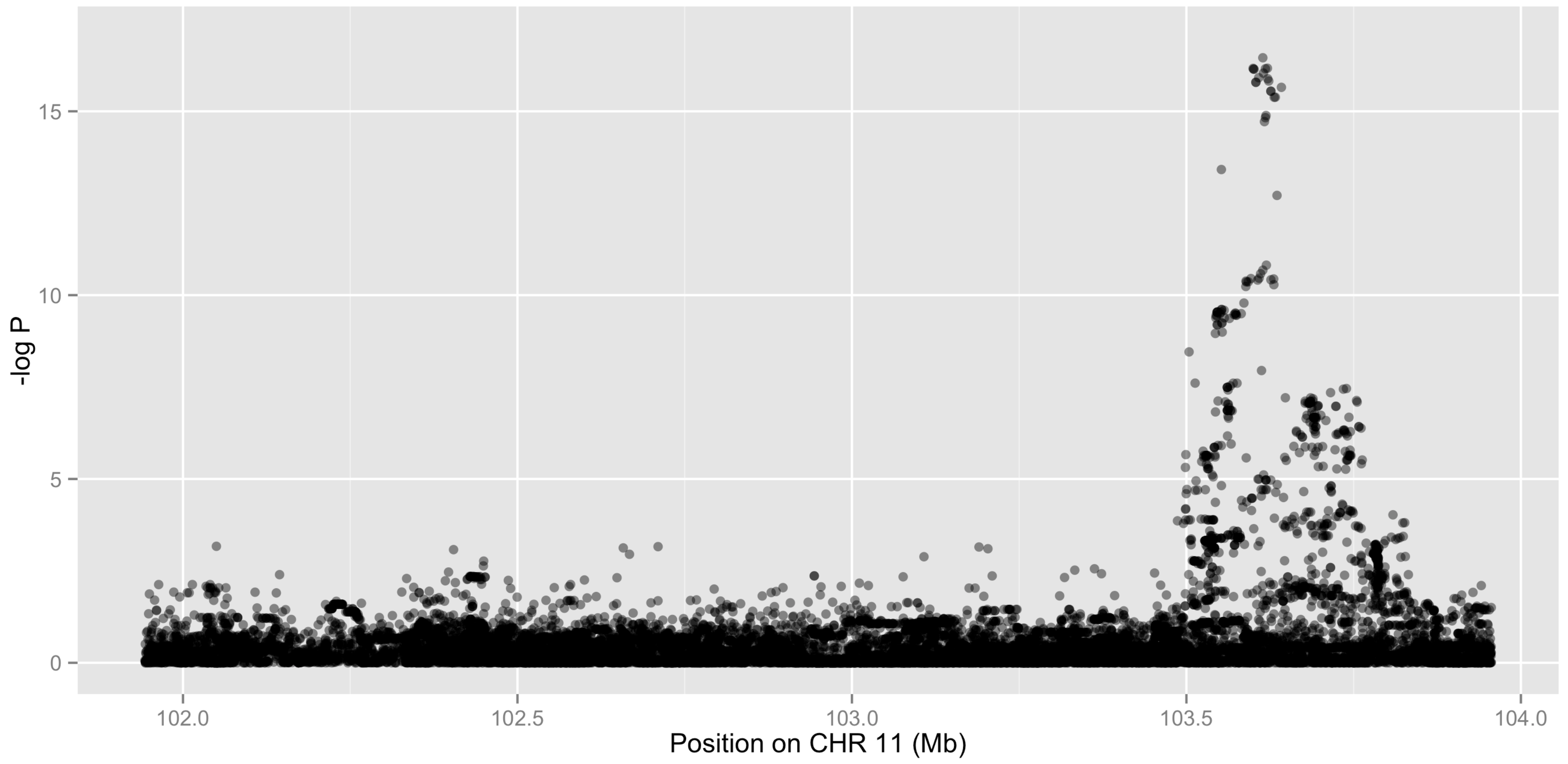

Contrast Manhattan plot of GCDH results

```
cdata = contrastData(gcdh_report$CHR1,
        gcdh_report$BP1,
        gcdh_report$P1,
        gcdh_report$P,
        gcdh_report$SNP1)
cplot = manhattanPlot(cdata)
ggsave("/tmp/cplot.png", cplot, width = 10, height = 5)
```

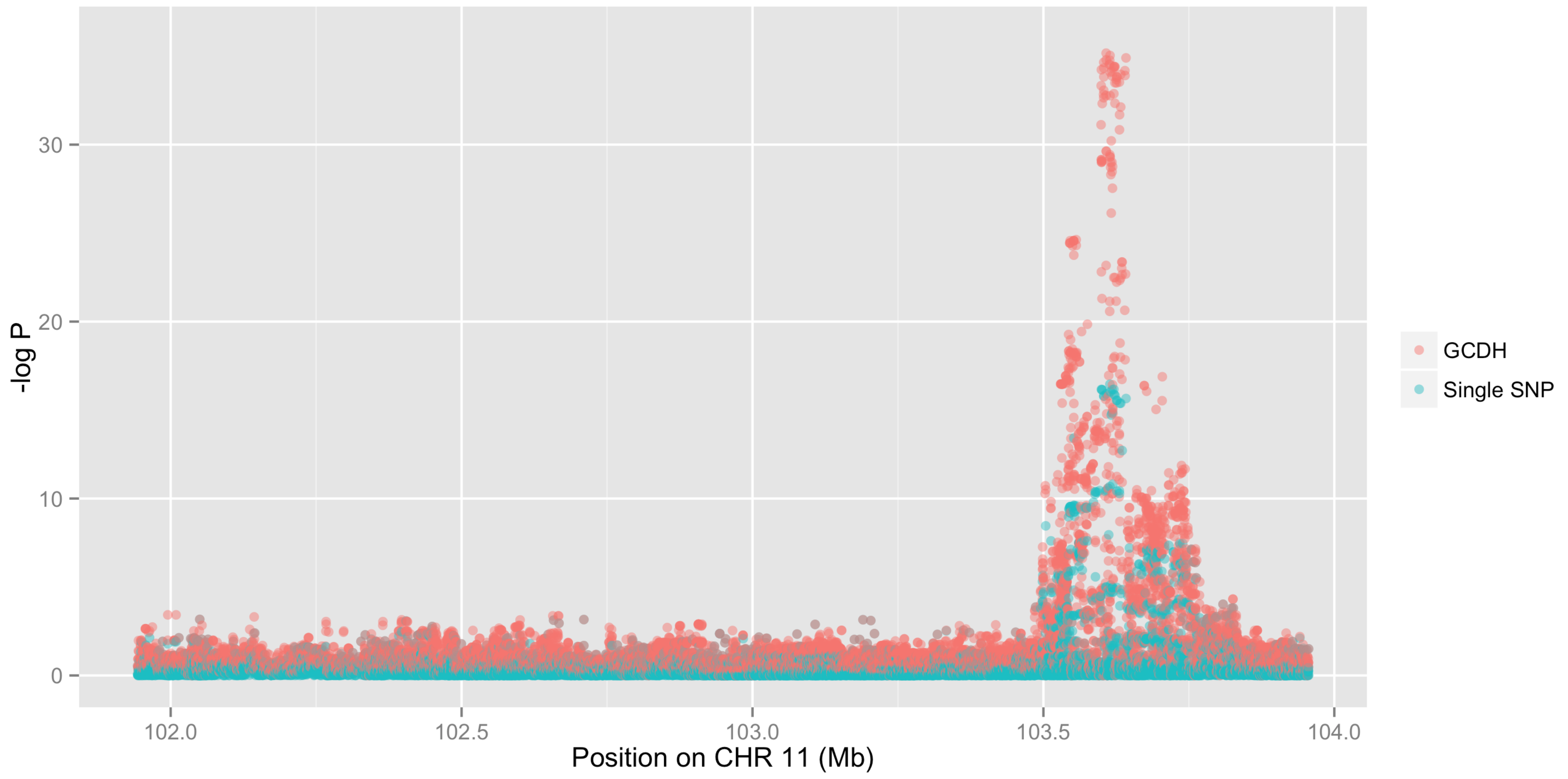
30
20
10
0
-log P
102.0
102.5
103.0
103.5
104.0
Position on CHR 11 (Mb)
GCDH
Single SNP
